# Counting nematodes made easy: leveraging AI-powered automation for enhanced efficiency and precision

**DOI:** 10.3389/fpls.2024.1349209

**Published:** 2024-06-26

**Authors:** Kanan K. Saikai, Trim Bresilla, Janne Kool, Norbert C. A. de Ruijter, Casper van Schaik, Misghina G. Teklu

**Affiliations:** ^1^ Agrosystems Research, Plant Science Group, Wageningen University and Research, Wageningen, Netherlands; ^2^ Ceradis B.V., Wageningen, Netherlands; ^3^ Laboratory of Cell and Developmental Biology, Department of Plant Sciences, Wageningen University and Research, Wageningen, Netherlands; ^4^ Laboratory of Nematology, Department of Plant Sciences, Wageningen University and Research, Wageningen, Netherlands

**Keywords:** nematode diagnostic, deep-learning, Meloidogyne, root-knot nematode, (CNN) convolutional neural network

## Abstract

Counting nematodes is a labor-intensive and time-consuming task, yet it is a pivotal step in various quantitative nematological studies; preparation of initial population densities and final population densities in pot, micro-plot and field trials for different objectives related to management including sampling and location of nematode infestation foci. Nematologists have long battled with the complexities of nematode counting, leading to several research initiatives aimed at automating this process. However, these research endeavors have primarily focused on identifying single-class objects within individual images. To enhance the practicality of this technology, there’s a pressing need for an algorithm that cannot only detect but also classify multiple classes of objects concurrently. This study endeavors to tackle this challenge by developing a user-friendly Graphical User Interface (GUI) that comprises multiple deep learning algorithms, allowing simultaneous recognition and categorization of nematode eggs and second stage juveniles of *Meloidogyne* spp. In total of 650 images for eggs and 1339 images for juveniles were generated using two distinct imaging systems, resulting in 8655 eggs and 4742 *Meloidogyne* juveniles annotated using bounding box and segmentation, respectively. The deep-learning models were developed by leveraging the Convolutional Neural Networks (CNNs) machine learning architecture known as YOLOv8x. Our results showed that the models correctly identified eggs as eggs and *Meloidogyne* juveniles as *Meloidogyne* juveniles in 94% and 93% of instances, respectively. The model demonstrated higher than 0.70 coefficient correlation between model predictions and observations on unseen images. Our study has showcased the potential utility of these models in practical applications for the future. The GUI is made freely available to the public through the author’s GitHub repository (https://github.com/bresilla/nematode_counting). While this study currently focuses on one genus, there are plans to expand the GUI’s capabilities to include other economically significant genera of plant parasitic nematodes. Achieving these objectives, including enhancing the models’ accuracy on different imaging systems, may necessitate collaboration among multiple nematology teams and laboratories, rather than being the work of a single entity. With the increasing interest among nematologists in harnessing machine learning, the authors are confident in the potential development of a universal automated nematode counting system accessible to all. This paper aims to serve as a framework and catalyst for initiating global collaboration toward this important goal.

## 1 Introduction

The process of nematode counting is both labor-intensive and time-consuming, yet it serves as a crucial step in numerous quantitative nematological studies, including the preparation of initial population and final population densities related to nematode management in pot, micro-plot and field trials (Barker and Campbell, 1981). Accurate results heavily depend on the expertise of taxonomy of nematodes of the individuals conducting this task. This study aims to streamline the nematode counting process for *Meloidogyne* spp. juveniles and eggs by implementing a deep learning algorithm for automation.

Traditionally, nematode counting has been a manual process (Hussey and Barker, 1973; Seinhorst, 1988). Following the extraction of nematodes from plant materials or soil, they are collected in a water suspension and subjected to counting. Prolonged storage in water suspension makes the task of counting and identifying nematodes progressively challenging, even for experienced nematologists, as nematode might die due to depletion of stored food. In some cases, all nematodes in the entire sample are counted, but this can be impractical, especially when dealing with a large number of samples or a high nematode density per sample, due to the considerable time required. Consequently, many laboratories opt to count nematodes by analyzing subsamples from the mother suspension, although this approach can introduce errors and diminish statistical accuracy when few nematodes are counted (Schomaker and Been, 1998). Furthermore, the manual nematode counting process is susceptible to inconsistency when carried out by different individuals or prone to errors that may arise when an individual spends an extended period behind a microscope.

Nematologists have long recognized the challenges associated with nematode counting, prompting a few research initiatives to automate the process. Been et al. (1996) introduced ANECS (Automatic NEmatode Counting System), a software program designed to count juveniles of *Globodera* spp in water suspension. While this method achieved high detection accuracy, its widespread adoption among nematologists was hindered by the need for specialized and expensive hardware and image analysis systems. In more recent years, Holladay et al. (2016) adapted ImageJ, an open-source image analysis software, to create a standard curve for automated nematode counting based on the black and white pixel sizes in individual images. However, this method is limited to samples containing a single species of similarly sized nematodes and excludes samples with soil and root debris. Given the recent advancements in artificial intelligence, these challenges encountered during automated nematode counting can now be effectively tackled.

Deep learning, a broader realm within machine learning encompassing various architectures like Convolutional Neural Networks (CNNs), has garnered significant research attention among nematologists, as indicated by recent studies (Akintayo et al., 2018; Chen et al., 2020, 2022; Kalwa et al., 2019; Uhlemann et al., 2020). The utilization of neural networks for computer-assisted nematode identification dates as early as 2000 when it was proposed by Diederich et al. (2000). CNNs, the focus of this study, are computer programs designed to replicate how our brains process visual information, proving highly effective in the analysis of biological images, such as microscope pictures. Notably, there are compelling examples of AI-driven approaches for automating nematode counting. In their work, Akintayo et al. (2018) applied a deep learning architecture originally designed for detecting rare objects in cluttered images to the task of identifying eggs of *Heterodera glycines*. They designed the Convolutional Selective Autoencoder (CSAE) architecture, which facilitated rapid detection, consistency, and accuracy in identifying nematode eggs amidst debris. Likewise, Kalwa et al. (2019) developed a modified version of the U-Net convolutional autoencoder model learning algorithm, specifically customized for detecting *H. glycines* eggs in purified samples. In contrast to typical approaches using microscopic images, their imaging systems employed a high-resolution scanner and a light-emitting diode (LED) to illuminate the processed sample flowing through a microfluidic flow chip, along with a CMOS image sensor. Both studies necessitated prior staining. In 2020, Chen et al. adapted the standard U-Net architecture to automate the counting of worm-shaped objects. Instead of employing the standard bounding box detection method used in other studies, they utilized the skeleton to address overlapping and curled nematodes. Their initial model was primarily assessed on *C. elegans*. Subsequently, in 2022, Chen et al. extended their work by developing a segmentation model for cyst detection in soil debris. This was accomplished by leveraging the standard U-Net architecture and ResNet architecture. While not focused on counting, Uhlemann et al. (2020) effectively employed CNNs to differentiate between three entomopathogenic nematode species within the same family. To select the most appropriate architecture for their study, the researchers initially screened 13 CNN architectures available in 2020. They ultimately decided to employ Xception due to its highest accuracy among the options. Additionally, Shabrina et al. (2023) devised a deep-learning model aimed at automatically identifying 11 different genera of plant-parasitic nematodes commonly found in Indonesia. Although their primary focus was classification rather than quantification like Uhlemann et al. (2020), they investigated four distinct architectures, ResNet101V2, CoAtNet-0, EfficientNetV2Bo, and EfficientNetV2M, across various augmentation processes. Their research culminated in the creation of a website capable of analyzing nematode images and providing genus-level identification.

While the aforementioned studies have demonstrated the effectiveness of deep learning for automating nematode counting, they have primarily been limited to single-class object identification within individual images. To make this technology more practical, there is a need for an algorithm capable of simultaneously detecting and classifying multiple objects, even when they cohabit with other objects that are confusing shapes and sizes, such as root and soil debris or non-target nematodes within individual samples. This study aims to address this challenge by developing a deep learning algorithm that can simultaneously identify and classify nematode eggs and juveniles of *Meloidogyne* spp. while distinguishing them from free-living nematodes and other clutter in the sample. The resulting algorithm will be shared as open-source software on GitHub for public use.

## 2 Materials and methods

### 2.1 Sample preparation

**Figure 1:**
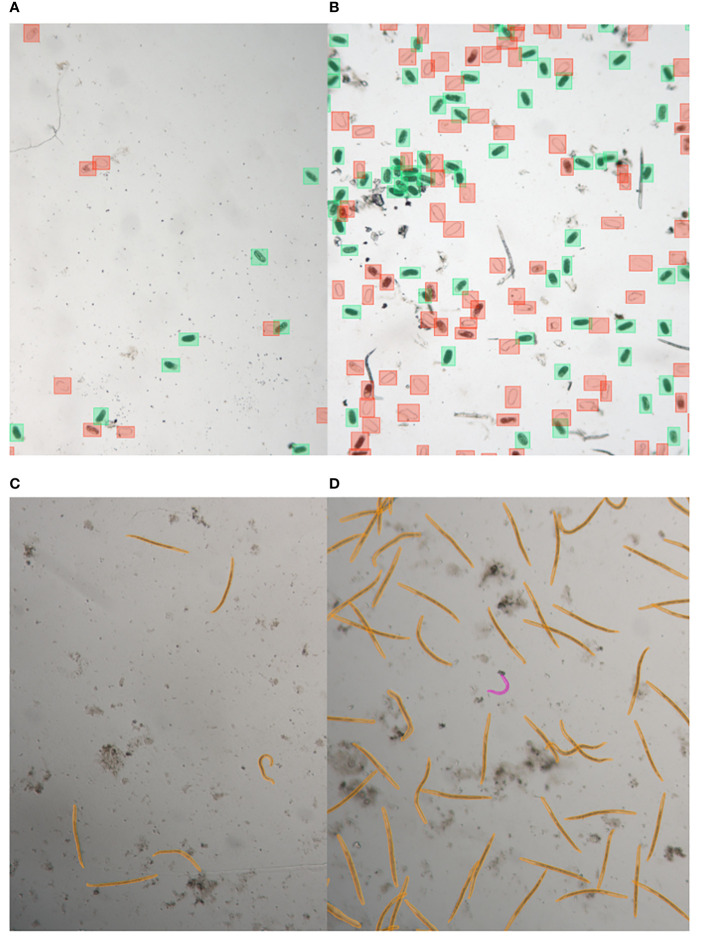
Illustrative samples of nematode egg and juvenile annotated images captured with LEITZ DM IRB. Images show eggs with low abundance **(A)** and high abundance **(B)**, annotated using bounding box annotations, as well as juveniles with low abundance **(C)** and high abundance **(D)**, annotated using segmentation.

Eggs and second stage juveniles (J2) of *Meloidogyne* spp (RKN). were acquired from cultures maintained on tomato plants by the Plant Science Group at Wageningen University & Research. Eggs were extracted from tomato roots using the bleach method (Hussey and Barker, 1973) and J2s were obtained by incubating 5-cm pieces of infected tomato roots in a mist chamber (Seinhorst, 1988). To create varying densities of nematodes in water suspension containing either eggs or J2s, we diluted the original suspensions. This allowed us to replicate scenarios with specimens in low abundance (non-overlapping specimens) to high density (commonly overlapping specimens) (**Figure 1**). The J2 suspensions occasionally contained free-living nematodes as contaminations. Before imaging, the J2 suspensions in a petri dish were subjected to a temperature of 40°C for a period ranging from 30 seconds to 1 minute. This heat treatment was employed to minimize their movement and facilitate the imaging process. For further imaging, each well of the CELLSTAR 24 Well Cell Culture Plate (Greiner Bio-One B.V., Alphen aan den Rijn, The Netherlands), was filled with 2ml of either the egg or J2 suspensions.

### 2.2 Image acquisition and annotation

The primary imaging tool for both eggs and J2s was the Leica Stellaris 5 Confocal LSM on a DMi8 microscope (Leica Microsystems, Wetzlar, Germany). Image acquisition was facilitated using LAS-X software V4.40 (Leica Microsystems) with LAS-X Navigator with Assay Editor to automatically visit any assigned location in Multiwell plates, resulting in a total of 600 images for eggs and 1289 images for J2s, which were automatically stitched (at 10% overlap) to display overviews of 24 well plates at a chosen resolution. To ensure image diversity for the algorithm development, two different magnifications and resolutions were employed at standard speed of 600 µm/s, using 400 Hz scan speed at 512 x 512 pixels. Eggs and juveniles were imaged with 5x (NA 0.15) or 10x (NA 0.40) objective. The bright field images used in this study were captured as full transmission images and do not display a confocal z-slice. To further enhance the variety of images for computer learning during the algorithm development process, another LEITZ DM IRB inverted microscope (Leica Microsystems, Wetzlar, Germany) was utilized. Image acquisition was facilitated using ZEISS ZEN lite software (Carl Zeiss NTS Ltd, Oberkochen, Germany) equipped with a Zeiss Axiocam-712 color camera. With a 10x objective (NA 0.22), this camera captured 50 images each for both eggs and juveniles for annotation. A confocal image can be adjusted to any pixel density compatible with the resolution provided by the objective’s numerical aperture (NA), while images captured using the LEITZ DM IRB system with the Axiocam 712 are set at a fixed, high pixel density. The acquisition methods differ significantly: the confocal acquires data pixel by pixel using the XY scanner, whereas the CMOS chip in the Axiocam 712 captures entire frames simultaneously.

Annotation for egg images involved two distinct object classes: dead egg (including eggshells) and nematode egg. These annotations were created using bounding box annotations (**Figures 1A, B**). Nematode eggs possess an oval morphology, which renders them amenable to straightforward detection techniques, such as bounding box annotations. Specifically, we employed LabelImg, an open-source graphical image annotation tool. Users can access this tool through its GitHub repository at [https://github.com/tzutalin/labelImg]. In the case of nematode juvenile images, we annotated two distinct object classes: root-knot nematode (RKN) and free-living nematode (FLN). This was achieved through segmentation annotation, utilizing Darwin V7 developed by V7 Labs (**Figures 1C, D**). The vermiform body structure of nematodes is well-suited for the use of segmentation-based methodologies. The segmentation algorithms are particularly effective in properly delineating objects with distinct shapes due to the well-adapted bounds of their thin forms. Further details about Darwin V7 can be found on the V7 Labs website: [https://www.v7labs.com]. A total of 8655 eggs and 5379 dead eggs were annotated from the egg images, along with 4742 RKNs and 1153 FLNs from the juvenile images.

### 2.3 Model development

#### 2.3.1 Environment setup

The training approach employed a resilient hardware setup. To facilitate expedited computations for deep learning, a specialized NVIDIA GTX 4080Ti Graphics Processing Unit (GPU) with a memory capacity of 12 GB GDDR6X was employed. The necessary computational assistance was facilitated by an Intel Core i9–10900K processor, which possesses a default clock speed of 3.7 GHz and is equipped with 10 cores. The efficiency of data administration and model optimization was enhanced by the system’s utilization of 64 GB of high-speed DDR4 RAM. Additionally, the inclusion of a 1 terabyte Solid-State Drive (SSD) facilitates expedited data retrieval and efficient storing of model checkpoints.

The training environment utilized Ubuntu 20.04 LTS, a widely-adopted and reliable Linux system renowned for its robustness in supporting deep learning activities. The utilization of GPU acceleration was achieved by the utilization of CUDA Toolkit 11.2, which was specifically designed for the NVIDIA GTX 4080ti GPU. The assurance of GPU compatibility was achieved with the installation of NVIDIA Driver version 465.19.01. Python 3.8.10 was utilized as the principal programming language to facilitate smooth connection with deep learning frameworks. Additionally, the OpenCV library version 4.5.3 was incorporated to enable sophisticated image processing operations.

The Mamba package manager, which serves as a viable alternative to Conda, was employed to handle the training environment. The rationale for this choice was rooted in the enhanced effectiveness of Mamba in handling package dependencies and resolving conflicts within the environment. The ‘yolo_env’ Conda environment was constructed using Python 3.8. The integration of Mamba into the installation process facilitated the design of the environment and the installation of packages, ensuring a configuration that was free from errors and efficient. Afterwards, the necessary software packages, such as OpenCV and other relevant components, were installed via Mamba in order to achieve a smooth integration of libraries inside the system. This approach not only facilitated expedited environment configuration, but also enhanced the reliability and replicability of the training pipeline.

#### 2.3.2 YOLOv8 model

YOLOv8, the most recent iteration of YOLO (You Only Look Once) object detection architecture as of January 10 in 2023, was chosen to build a deep learning model for the classification and detection of nematode eggs and juveniles. YOLOv8 is a highly adaptable solution that excels in many tasks related to object recognition and picture segmentation. It effectively combines attributes such as speed, accuracy, and user-friendliness, resulting in a successful and efficient approach. The versatility of the system is demonstrated by its capacity to handle big datasets, and its effectiveness across a range of hardware platforms, including both CPUs and GPUs, is noteworthy. YOLOv8 stands out for its superior performance in terms of both accuracy and execution speed compared to other models. To set up the latest version of the YOLOv8 library in a Python environment, the “ultralytics” package was imported, as detailed in https://yolov8.com/. The Ultralytic repository provides a comprehensive description of the YOLOv8 model architecture.

In a nutshell, it’s important to note that YOLOv8’s anchor-free detection method improves its ability to handle a wide range of object sizes and shapes, all while simplifying the training process. The anchor-free detection in YOLOv8, predicting object centers directly, bypassing the need for predefined anchor boxes. This improves flexibility and efficiency, eliminating manual selection challenges and potential suboptimal results. Additional change in the YOLOv8 architecture which is relevant to our models is the replacement of C3 with C2f in the backbone, which altered the structure. Both C3 and C2f refer to distinct layers within the neural network architecture utilized for object detection. C3 represents a convolutional layer in the YOLO network, which comprises several layers followed by fully connected layers. In contrast, C2f serves as the fully connected layer succeeding the convolutional layers in YOLO’s architecture. The C2f layer is responsible for processing the high-level features extracted by the convolutional layers to generate the final predictions. This alternation in the structure includes switching a 3x3 for the initial 6x6 convolution in the stem. In C2f, outputs from the Bottleneck are integrated, unlike in C3 where only the final output is used. YOLOv8 still maintains YOLOv5’s Bottleneck structure, with the first convolution shifting from 1x1 to 3x3, aligning with the REsNEt block defined in 2015.

We employed the YOLOv8 Extra Large (YOLOv8x) model, which is the most precise but also the slowest among the five YOLOv8 models currently accessible. We used the default settings for both the convolutional layers and hyperparameters. The annotation data was divided into training and validation sets, with 6800 eggs and 4140 dead eggs in the training set, and 1855 eggs and 1239 dead eggs in the validation set, and 3840 RKN and 897 FLN in the training set and 902 RKN and 256 FLN in the validation set. The model’s iterations were halted when the mean Average Precision at a 50% Intersection over Union (IoU) threshold for bounding boxes (metrics/mAP50(B)) reached a plateau, while the loss function indicated that the model was learning. IoU quantifies the overlap between predicted and ground truth bounding boxes, with values ranging from 0 to 1; 0 indicating no overlap and 1 indicating perfect overlap. The loss function evaluates the disparity between a model’s predicted output and the actual target output, providing a measure of its performance on a given task. Minimizing this loss function during training aims to enhance accuracy in object detection. This step was taken to prevent overfitting. The source code for developing our models is available to readers on the author’s GitHub repository (https://github.com/bresilla/nematode_counting).

#### 2.3.3 GUI development

A graphical user interface (GUI) was created to seamlessly integrate segmentation and detection models for nematode eggs and juveniles. This GUI was built using Python and leveraged the “Tkinter” packages. The specific capabilities and features of the GUI are elaborated in the results section. The source code for the development of our GUI can be accessed by readers on the author’s GitHub repository (https://github.com/bresilla/nematode_counting).

**Figure 2:**
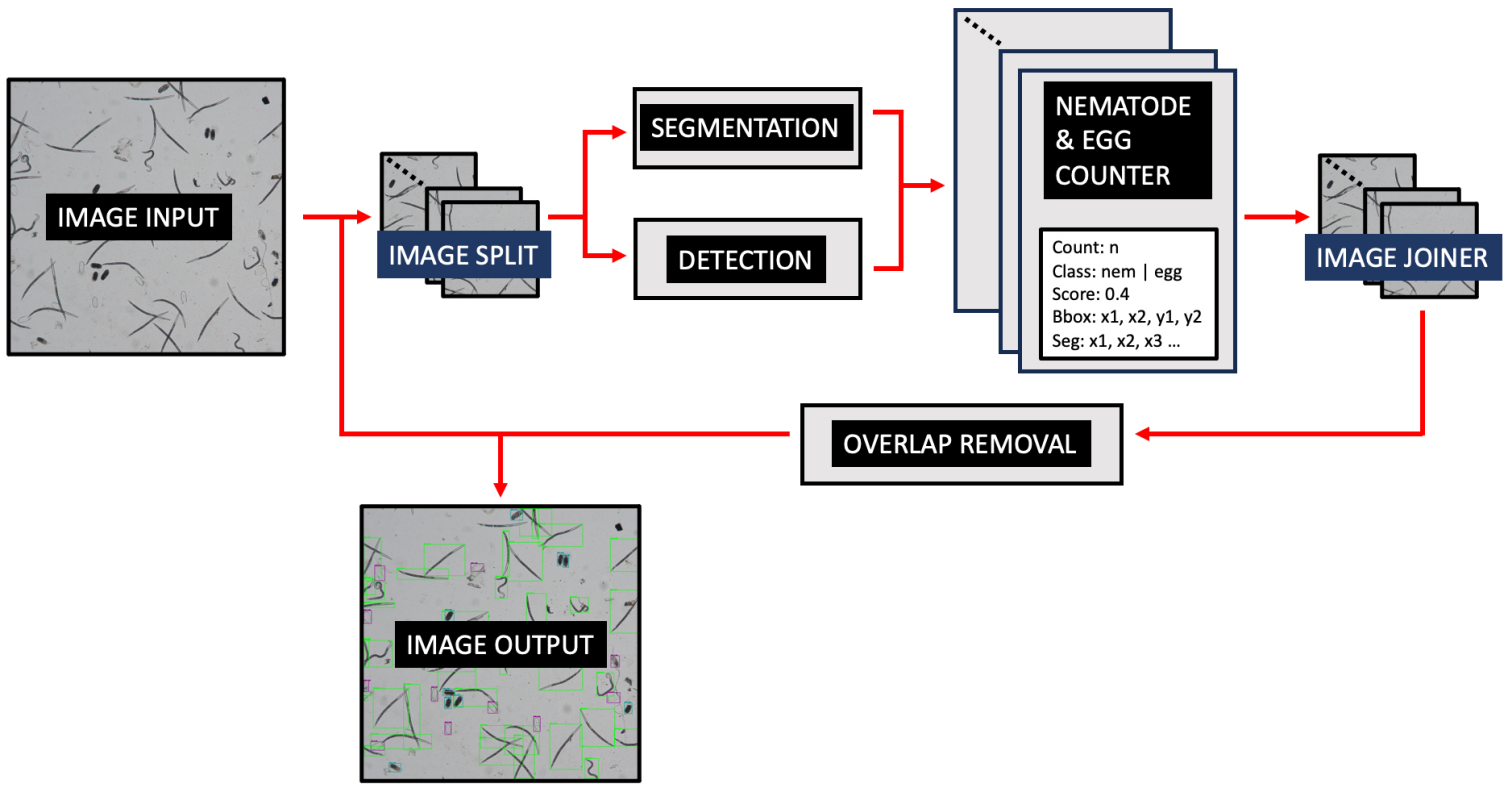
The operational workflow of SEGNEMA.

**Figure 2** demonstrates the operational workflow of SEGNEMA. Following the user’s selection of either a singular image or a batch of images, the application proceeds to partition the designated image into multiple smaller segments. The user retains the autonomy to specify the level of segmentation or opt for an undivided representation. Subsequent to this, the segmented images undergo simultaneous processing by two distinct models: the nematode detection model, responsible for segmentation, and the egg detection model, tasked with generating bounding boxes. Both models result in outputs detailing the quantity of detected and segmented objects. The segmented images are subsequently consolidated, and comprehensive labels, encompassing both detections and segmentations, are embedded. Furthermore, an independent module examines the results, probing for potential overlaps between segmentations and detections. To mitigate potential anomalies, such as duplicate edge detections, a post-consolidation step is employed to rectify redundant counts. This de-duplication process utilizes Intersection over Union (IoU) metrics. IoU measures the overlap between two bounding boxes drawn around detected objects, calculated as the area of overlap divided by the area of union between the two bounding boxes. By setting a threshold for IoU, redundant or overlapping detections can be effectively removed, thereby enhancing the accuracy and efficiency of object detection systems. Additionally, instances wherein an egg is erroneously identified as a nematode are addressed, with any overlaps exceeding the threshold of 60% leading to the elimination of the misidentified egg.

### 2.4 Inference on unseen images

The performance of GUI SEGNEMA was evaluated using a dataset of previously unseen images that included both eggs and juveniles. In total, 100 images were captured, with 50 from the Stellaris 5 Confocal LSM and 50 from the LEITZ DM IRB microscope, each taken at a magnification of 10x. Before being processed by the trained model, an expert conducted object counting for each class within individual images. To evaluate the model’s accuracy, the object count results obtained through the trained model were compared with those determined by the expert. In this evaluation, we examined the relationship between the two counting approaches by calculating the correlation coefficient and analyzing the discrepancies (residuals) between them. Furthermore, linear regression was conducted to assess a linear relationship between the observation and the model prediction for the classes of eggs and J2s. Given the premise that no objects should be detected in the absence of class objects within the images, we set the intercept to zero. This decision was made after conducting additional test runs of linear regression with an intercept and verifying that it was not significantly different from zero. The analyses mentioned earlier were carried out using R version 4.2.2, along with the default library.

Additionally, 10 well overviews from a 24-multiwell plate were made by stitching images with the Stellaris 5 Confocal LSM at a speed of 600 µm/s at a magnification of 10x. Each well contained 2ml of a water with both nematode eggs and juveniles. All images being auto-captured with 10% overlap and stitched by the LAS-X software V4.40, were processed with SEGNEMA to assess the model’s performance. The adjacent images used in the stitching process overlap by 10% of their width and height. This overlap helps in ensuring smooth transitions between the images when they are stitched together, reducing the chances of visible seams or discontinuities in the final stitched image. After being processed by the GUI, an expert performed object counting on the same 2ml of the test suspension using the conventional method of taking aliquot subsamples. In this method, the nematode eggs and juveniles were individually counted in one milliliter of the suspension in four repetitions after diluting to in total of 10ml with water, and the average count was used to determine the total number of test objects in the entire suspension volume. Similarly, to the aforementioned inference images, the relationship between the two counting approaches was analyzed by calculating the correlation coefficient and the residuals to estimate the discrepancy between the two approaches.

## 3 Results

### 3.1 Model trained for eggs

**Figure 3:**
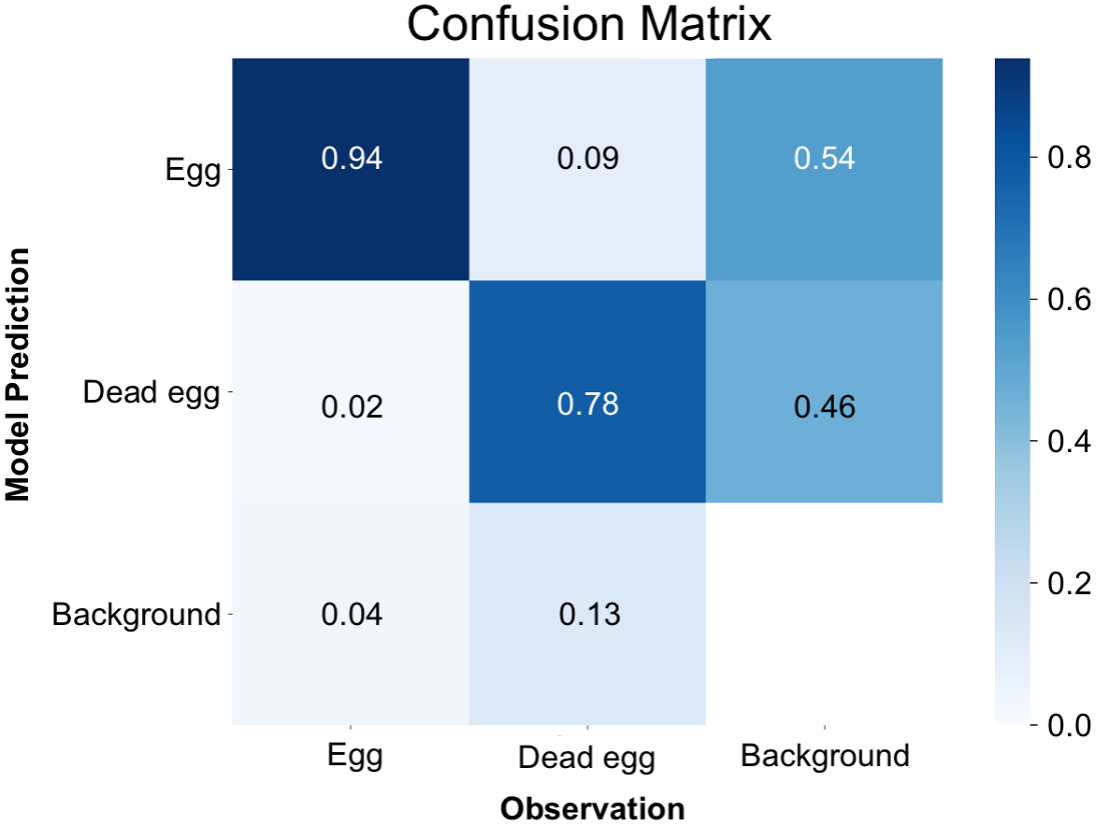
Confusion matrix for the detection of nematode eggs using the YOLOv8x deep-learning model.

By the time it reached the 500^th^ iteration, the model achieved its highest level of accuracy. This was evident through the mean Average Precision at a 50% Intersection over Union (IoU) threshold for bounding boxes (metrics/mAP50(B)) reaching to 0.86, which serves as a measure of the overall quality of object detection (**Supplementary Figure 1A**). Furthermore, the box loss score suggested that the trained model was still in the midst of learning, confirming that overfitting had not occurred yet. (**Supplementary Figure 1B**). Examining the confusion matrix (**Figure 3**), it was found that 94% of instances correctly identified eggs as eggs, and 78% accurately classified dead eggs as dead eggs. Conversely, there was a 2% error rate where instances mistakenly categorized eggs as dead eggs, and a 9% error rate where they erroneously labeled dead eggs as eggs. The model exhibited a failure to detect eggs in 4% of instances and had a similar failure rate of 13% for dead eggs. In specific cases, the model also mistakenly labeled background as one of the two classes, with an 54% occurrence for egg and a 46% occurrence for dead egg, respectively.

### 3.2 Model trained for second stage juveniles of *Meloidogyne* spp.

**Figure 4:**
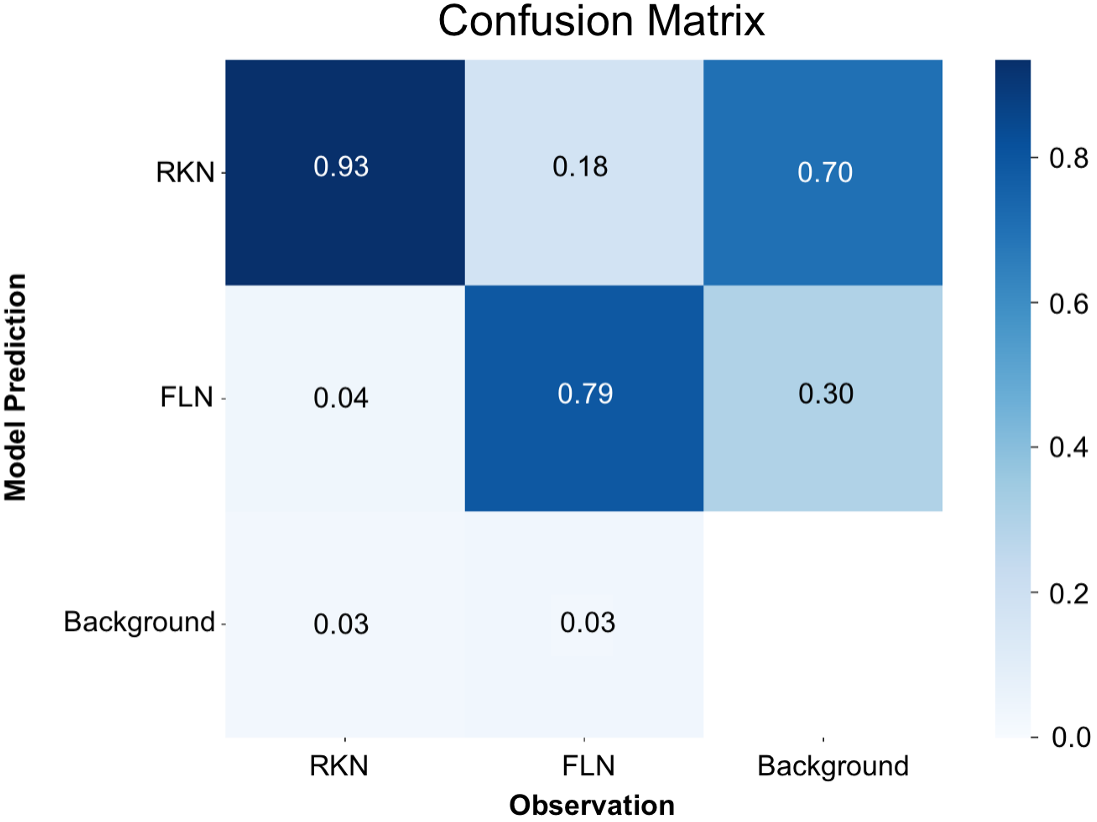
Confusion matrix for the detection of nematode juveniles using the YOLOv8x deep-learning model.

In a manner akin to the trained model for eggs, mAP50(M), which is similar to mAP50(B) but for segmentation, reached its zenith at 0.87 around the 200th iteration (**Supplementary Figure 2A**). Both the mAP50(M) and box loss score affirmed that the model attained the utmost level of accuracy without the risk of overfitting problem (**Supplementary Figure 2B**). Analysis of the confusion matrix (**Figure 4**) revealed that J2s of RKN were correctly identified as RKN in 93% of cases, and FLN were accurately classified as FLN in 79% of instances. Conversely, there was a 4% misclassification rate instances where J2s of RKN were mistakenly categorized as FLN, and 18% misclassification rate where FLN instances were erroneously labeled as J2s of RKN. Furthermore, the model encountered a failure to detect 3% of instances for J2s of RKN and 3% for FLN. In certain scenarios, the model also made the mistake of mislabeling the background as one of the two classes. When it occurs, 70% of such instances were for J2s of RKN and the remaining 30% were for FLN.

### 3.3 Instructions for GUI SEGNEMA

**Figure 5:**
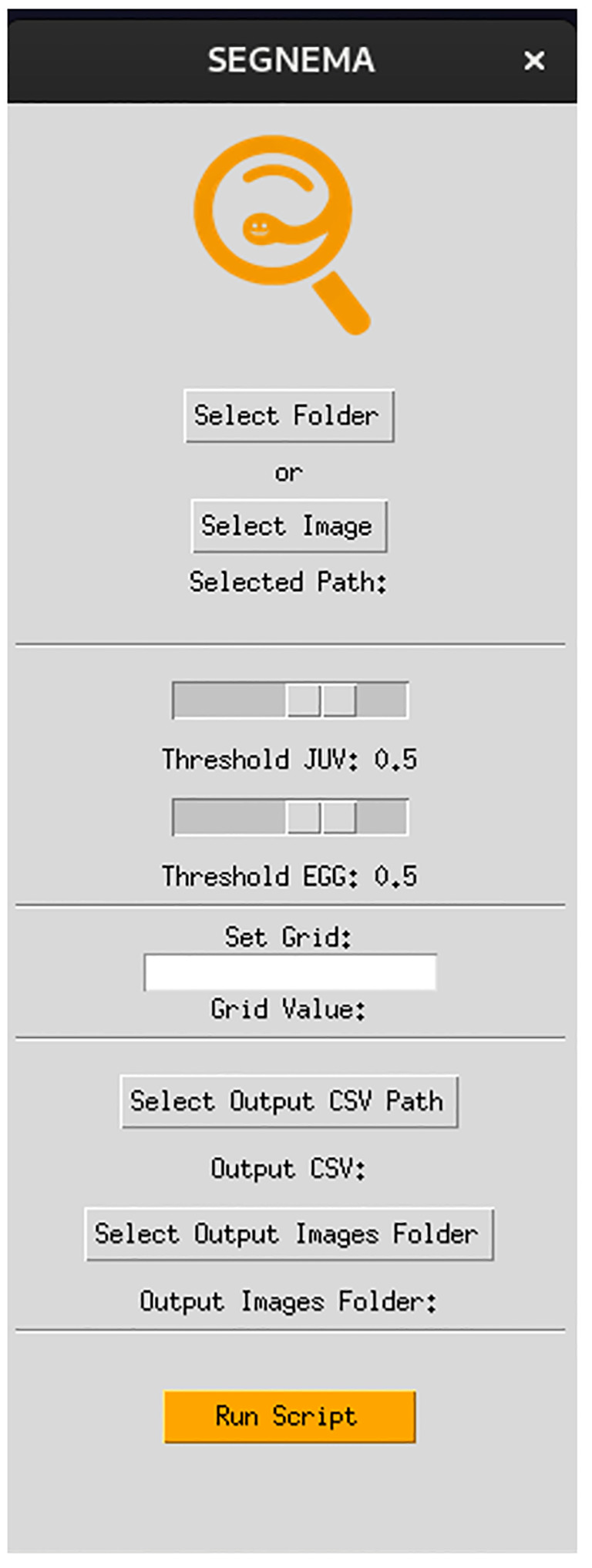
The interface of the GUI, SEGNEMA, which comprising the both nematode egg and juvenile detection models using YOLOv8x deep-learning models.

**Figure 6:**
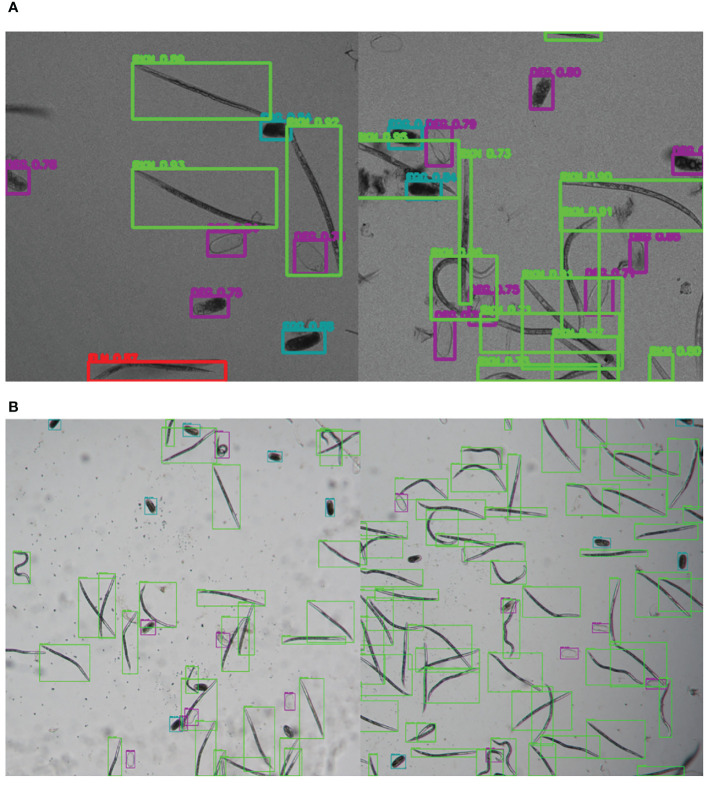
Illustrative samples of the output images of SEGNEMA, displaying the location of each class object with bounding box and their corresponding detection probability values, on the input images captured by Stellaris 5 Confocal LSM **(A)** and LEITZ DM IRB **(B)**.

The GUI, named SEGNEMA, was developed with individual models trained for egg and juvenile detection and classification (**Figure 5**). To use this GUI, the user system must meet certain environment requirements; The GUI itself doesn’t need any special requirements apart from a Python environment. However, to run the models, the computer needs to be set up to run neural networks. Specifically, it needs Pytorch installed. All the requirements and setup guide are within the shared link for the code (https://github.com/bresilla/nematode_counting). Users are prompted to make a choice between selecting a folder or an image for analysis through the user interface, specifying the file path accordingly. The interface offers additional options, such as “Threshold” to establish a detection accuracy threshold for each juvenile (“Threshold JUV”) and egg model (“Threshold EGG”), which are set at 50% as default. Users are responsible for adjusting the thresholds through trial and error to achieve optimal detection performance. This can be visually assessed by examining the location of each class object with its bounding box and the associated detection probability values when the models are executed using images captured with a new imaging system. The “Set Grids” option to partition an image into selected grid sizes. Furthermore, users need to define the output file path for a CSV file, which contains the numbers of computer-detected objects for each class, and for output images that display bounding boxes indicating the object locations within the image, as exemplified in **Figure 6**.

### 3.4 Inference for unseen images

**Figure 7:**
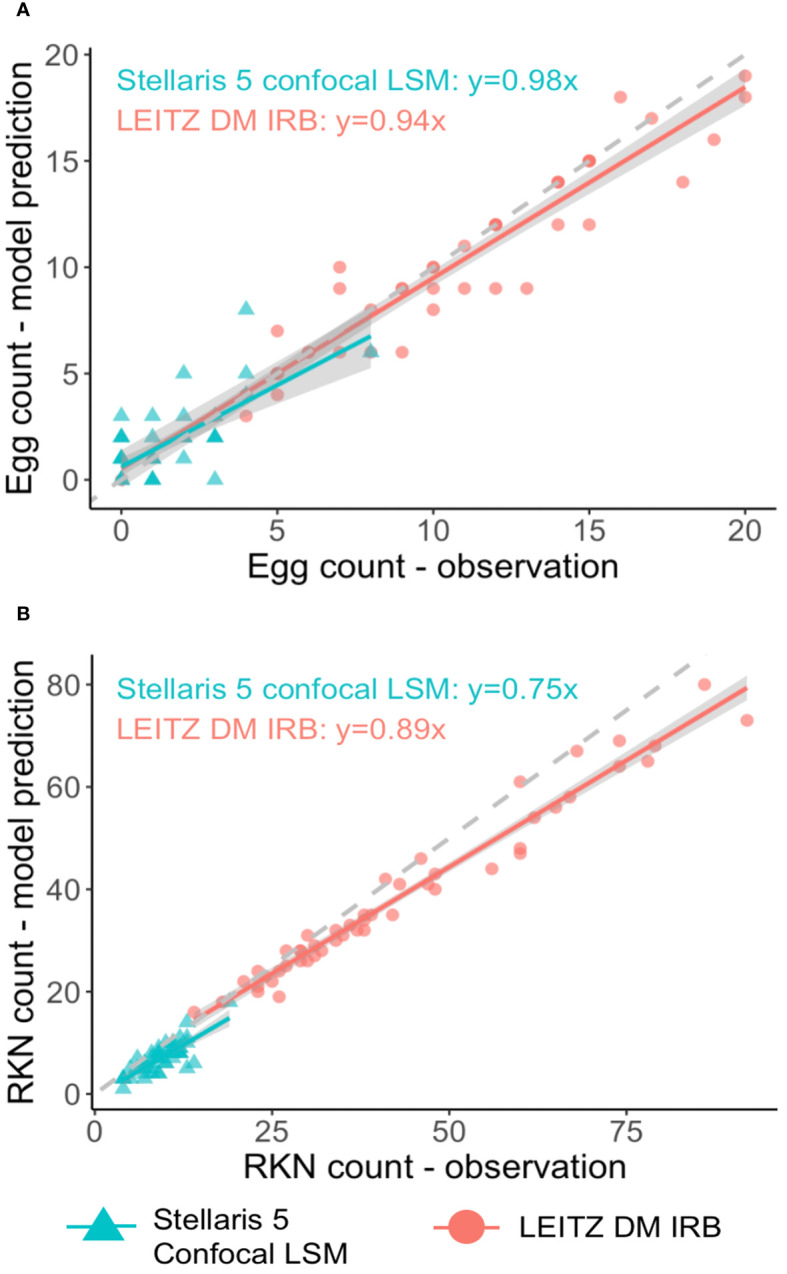
Linear regression analysis between model predictions and observation for nematode eggs **(A)** and *Meloidogyne* J2s **(B)** for two imaging systems.

The SEGNEMA GUI was used to perform inference on previously unseen images containing both nematode eggs and juveniles. The predefined thresholds were configured at 0.5 and 0.75 for juveniles, and 0.5 and 0.45 for eggs, specifically for the Stellaris 5 Confocal LSM and LEITZ DM IRB images, respectively. It normally took approximately 4 to 5 seconds to process a single image with the environment facilitated in this study. The model’s performance resulted in correlation coefficient values of 0.81 and 0.98 for J2s of RKNs, and 0.72 and 0.96 for eggs in the Stellaris 5 Confocal LSM and LEITZ DM IRB images, respectively (**Table 1**). The model performed well for eggs and J2s, as evidenced by the results in **Table 1**, even in scenarios where the samples were densely populated with objects (**Figure 7**). The model demonstrated the capability to differentiate between overlapped nematodes when their heads were oriented in different directions, effectively treating them as distinct entities. Moreover, the models exhibited successful object detection, even when the objects were only partially visible within the image. However, the model exhibits a tendency to encounter difficulties when processing irregularly shaped objects, such as nematodes forming spiral patterns or when two objects overlap, giving the impression of a single entity. On occasion, the model may mistakenly identify fibers present in the samples or plate scratches as nematodes, particularly categorizing them as free-living nematodes. Additionally, when nematodes adopt a curled configuration, resembling a round shape, the model tends to struggle in distinguishing whether it is a nematode or an egg. The linear regression models showed that the predicted number of eggs could be obtained by multiplying the manual counting (observation) by 0.98 for Stellaris 5 Confocal LSM and 0.94 for LEITZ DM IRB (*P*-value < 0.01). Similarly, the predicted number of J2s could be obtained by multiplying the manual counting by 0.75 and 0.89 for Stellaris Confocal LSM and LEITZ DM IRB, respectively (*P*-value < 0.01).

**Table 1:** Correlation coefficients, mean, and median of residuals (the number of observed instances in an image - the number of model-predicted instances in an image) for each class taken by two different imaging systems.

Image system	Class	Coefficient Correlation	Residual mean*	Residual median
Stellaris 5 Confocal LSM	Egg	0.72	-0.32	0
	Dead-egg	0.71	2.32	2
	RKN	0.81	2.32	2
	FLN	0.34	-2.04	-1.5
LEITZ DM IRB	Egg	0.96	0.52	0
	Dead-egg	0.96	-0.82	0
	RKN	0.98	4.44	4
	FLN	0.10	0	0

*Residual = (observation)-(model prediction).

**Figure 8:**
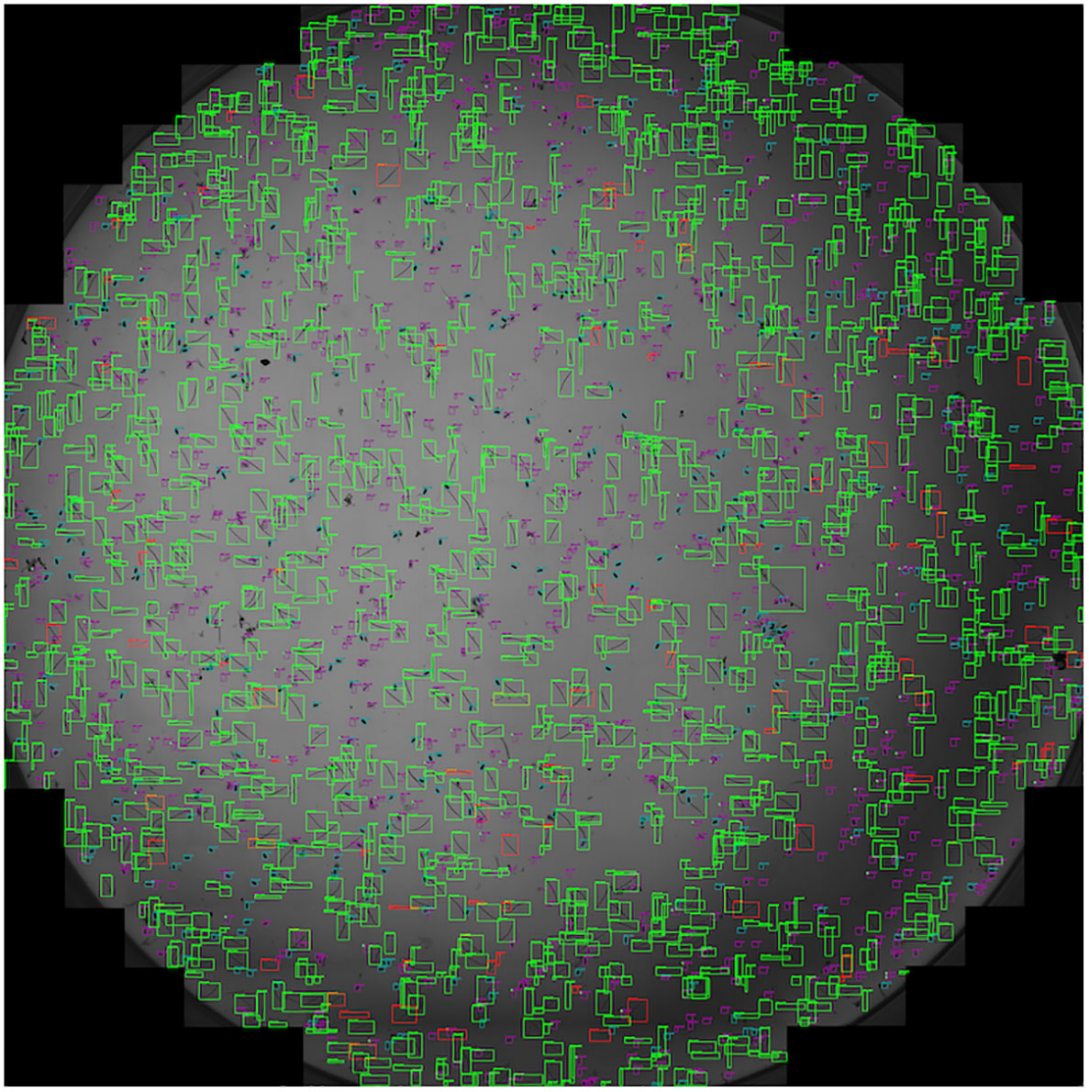
Illustrative sample of the output image of SEGNEMA, displaying the location of each class object with bounding box on the stitched input images captured by Stellaris 5 Confocal LSM.

Furthermore, the performance of SEGNEMA was assessed on 10 stitched images of an entire well that contained 1 ml of nematode suspension captured using Stellaris 5 Confocal LSM (**Figure 8**). The alteration made from the aforementioned configuration for the stitched images involved setting the grid size to 10x10 (Grid Size: 10x10). This adjustment allowed for the segmentation of a large-scale image, such as a 69.7MB stitched image, into a 10x10 grid. Consequently, this segmentation enabled processing by SEGNEMA. As per the conventional counting method, the counts of 10 stitched images for juveniles varied between 547 and 1553, while the counts for eggs ranged from 168 to 455. In contrast, using SEGNEMA, the counts of 10 stitched images for juveniles ranged from 492 to 1520, and for eggs, they ranged between 182 and 432. The correlation coefficients between the traditional nematode counting using subsampled aliquots and the counts produced by SEGNEMA were 0.99 for RKNs and 0.98 for eggs. The average and median values of the residuals for the J2s of RKN were 42.5 and 38.5, respectively. Similarly, for eggs, the average and middle values were -2.6 and -2.25, respectively. SEGNEMA processed 10 stitched images in about 3.5 minutes, while performing the same task manually took around 2.5 hours.

## 4 Discussion

Our research showcased the effectiveness of the trained models, which attained a detection accuracy of over 90% for both life stages of *Meloidogyne* spp. eggs and juveniles, a genus known for its economic importance in various crops (Jones et al., 2013). To make these models easily accessible, we have created an open-source application called SEGNEMA, which can be found on the author’s GitHub account (https://github.com/bresilla/nematode_counting). SEGNEMA enables the simultaneous detection and classification of nematodes and eggs. In the future, additional models can be independently created for different genera, including cyst nematodes like *Globodera* spp. and *Heterodera* spp., as well as *Pratylenchus* spp. These models can then be seamlessly incorporated into the same GUI through the use of transfer learning techniques.

While our models have exhibited high accuracy in detecting and classifying objects, they have encountered challenges that are commonly reported in similar studies, as noted by Akintayo et al. (2018), Been et al. (1996), and Chen et al. (2022). Like the models developed by Akintayo et al. (2018) and Been et al. (1996), our models faced difficulties in distinguishing overlapped objects as distinct entities and in discerning nematodes from organic debris within samples. Furthermore, our models exhibited reduced detection accuracy when it came to FLN compared to the other class objects in this study. This can be attributed to both the limited number of instances for this particular class and the diverse range of lengths and shapes that FLN can assume. Nevertheless, these challenges can be mitigated by incorporating additional images that specifically address these conditions. This study has introduced a framework that can serve as a universal automation solution for nematode detection amidst multiple objects. However, it’s crucial to recognize that no model is flawless, and ongoing improvements in object detection and classification are essential. Therefore, developers and users alike should remain vigilant in identifying scenarios where models may fall short.

In addressing some of the challenges encountered during the development of our model for this study, we experimented with various techniques. Initially, we employed bounding boxes for annotating nematodes, a method commonly used in object detection (Huang et al., 2016), as seen in the work by Akintayo et al. (2018). However, we found that this approach did not yield the desired level of detection accuracy, especially when dealing with objects like nematodes that exhibit diverse shapes, overlap with each other, or are surrounded by debris in the sample. Subsequently, after the unsuccessful attempt with bounding boxes, we explored alternative annotation methods and ultimately concluded that segmentation offered the highest level of detection accuracy. We also investigated the utilization of the skeleton, referred to as key-point detection, as suggested in the study by Chen et al. (2020), which demonstrated successful detection of intertwined and overlapping worm-shaped objects. However, for the small preliminary dataset we used, segmentation proved to be more effective in distinguishing RKN J2s from FLN. Additionally, it’s worth mentioning that even with 10 key-points required to annotate an object, the process was quite labor-intensive and frequently necessitated additional key-points, particularly when annotating curled nematodes. With Darwin V7, the annotation system we employed, which allows for auto-segmentation of objects, facilitated the generation of the large annotated dataset in our study. The utilization of segmentation has also been applied successfully in other studies, such as the detection and phenotyping of cysts in samples with debris, as demonstrated by Chen et al. (2022). Segmentation proved to be a valuable technique for annotating nematodes with diverse shapes, leading to noticeable enhancements in the model’s ability to correctly identify curled nematodes as nematodes, rather than misclassifying them as eggs. However, it’s important to note that further improvement in this regard would benefit from an increased dataset comprising more images depicting such cases.

Moreover, we observed a notable enhancement in our models’ object detection performance across different images and a notable increase in the mAP50 after integrating annotated images obtained from an additional microscope system. Specifically, the mAP50 for the egg model improved from 0.79 to 0.86, while for the juvenile model, it increased from 0.82 to 0.87. Prior to the inclusion of annotated images from the LEITZ DM IRB microscope, our models exhibited high accuracy in object detection primarily on images taken by the Stellaris 5 Confocal LSM system only. However, this performance improved considerably, as illustrated in **Table 1** for the final model. Despite the fact that we utilized only two imaging systems in this study, this expansion in the variety of images has a profound impact on the models’ ability to achieve accurate detection. As machine learning models benefit from exposure to a wider array of images, this development translates to enhanced detection accuracy across different imaging systems, beyond those used in this particular study.

An issue previously raised in the context of automated counting systems was their reliance on specialized and costly hardware and image analysis systems, as highlighted by Been et al. (1996). In our study, we addressed this concern by creating a user-friendly GUI called SEGNEMA, which is open source and freely accessible (https://github.com/bresilla/nematode_counting). Once the optimal detection threshold is determined for eggs and juveniles, users should be able to maintain it consistently for the same imaging system, streamlining the usability of SEGNEMA for their specific needs. While SEGNEMA does necessitate a computer environment equipped with substantial graphics processing units (GPUs), this requirement remains reasonably accessible to a wide range of users. The processing time per image using SEGNEMA was typically around 4 to 5 seconds for our images, though this duration may vary depending on the image size. This provides a significantly faster alternative to manual labor for nematode counting, contributing to increased efficiency and productivity in the analysis process. For example, at a magnification of x10 and a speed of 600 µm/s with the Stellaris 5 Confocal LSM, capturing 10 stitched images required 58 minutes. Adjusting the speed can enhance image resolution, but this comes at the cost of a longer image capture time. It’s essential for readers to recognize the trade-off between efficiency (speed) and reliability (resolution). Even when considering the processing time using SEGNEMA, the total time is still less than half of what is typically needed for the conventional nematode counting method involving aliquot subsampling. Moreover, because the imaging system’s stitching process is automated, the waiting period during image acquisition can be utilized for other tasks. Crucially, SEGNEMA ensures consistency in nematode counting, uninfluenced by variations in conditions, unlike manual counting methods.

Our research has effectively demonstrated the promising practical applications of these models, particularly through the user-friendly GUI that enables simultaneous detection and classification of nematodes. Presently, this GUI is tailored for nematode juveniles and eggs, but it possesses significant potential for broader applications across various nematode genera and in different media beyond aqueous solutions. The potential of acquiring fluorescent images simultaneous with the bright field images obtained from the confocal microscope is large and opens doors to automated multiplex (high content) acquisition with no time loss. Without any incubation or staining the autofluorescent images or spectra of nematodes can e.g. provide information on their viability (Forge and MacGuidwin, 1989), and probes can be used to add further specificity to the nematode discrimination.

It’s also crucial to address the challenges identified in our study and similar research endeavors. However, tackling these challenges cannot be the sole responsibility of a single laboratory or research institution. Instead, it requires a collaborative effort, bringing together expertise from both nematologists and AI researchers across multiple organizations and research groups. For our GUI to be thorough in nematode detection and diagnostics, it’s imperative to acquire a wider array of images captured through diverse imaging systems and develop models for different genera. Achieving this goal necessitates enhanced collaboration with fellow nematologists to access their image collections and tap into their expertise in nematode diagnostics. Given the growing interest among nematologists in harnessing the potential of machine learning, we are confident that the development of a universal automated nematode counting system accessible to everyone is within reach. The authors of this paper hope that it serves as a framework and catalyst for initiating global collaboration toward this important goal.

## Author’s note

The subsequent authors following the first author are arranged in alphabetical order.

## Data availability statement

The raw data used to develop models presented in this article will be made available by the authors, without reservation.

## Author contributions

KS: Writing – original draft, Writing – review & editing. TB: Writing – original draft, Writing – review & editing. JK: Writing – review & editing. NdR: Writing – review & editing. CvS: Writing – review & editing. MT: Writing – review & editing.

## Supplementary material

Supplementary Figure 1 Scores of mAP50(B) **(A)** and Box Loss **(B)** over iterations for the YOLOv8x deep-learning model for nematode egg detection.

Supplementary Figure 2 Scores of mAP50(M) **(A)** and Box Loss **(B)** over iterations for the YOLOv8x deep-learning model for nematode juvenile detection.

## References

[B1] Akintayo A. Tylka G. L. Singh A. K. Ganapathysubramanian B. Singh A. Sarkar S. (2018). A Deep learning framework to discern and count microscopic nematode eggs. Sci. Rep. 8, 9145. doi: 10.1038/s41598-018-27272-w 29904135 PMC6002363

[B2] Barker K. Campbell C. (1981). “Sampling nematode populations,” in Plant parasitic nematodes. Eds. Zuckerman B. M. Rohde R. A. (Academic Press, New York), 451–474.

[B3] Been T. H. Meijer E. M. J. Beniers A. E. Knol J. W. (1996). Using image analysis for counting larvae of potato cyst nematodes (*Globodera* spp.). Fundam. Appl. Nematol. 19, 297–304.

[B4] Chen L. Daub M. Luigs H.-G. Jansen M. Strauch M. Merhof D. (2022). High-throughput phenotyping of nematode cysts. Front. Plant Sci. 13, 965254. doi: 10.3389/fpls.2022.965254 36186075 PMC9515587

[B5] Chen L. Strauch M. Daub M. Jiang X. Jansen M. Luigs H.-G.. (2020). “A CNN framework based on line annotations for detecting nematodes in microscopic images,” in 2020 IEEE 17th International Symposium on Biomedical Imaging (ISBI), USA. 508–512 (IEEE, IA). doi: 10.1109/ISBI45749.2020.9098465

[B6] Diederich J. Fortuner R. Milton J. (2000). Genisys and computer-assisted identification of nematodes. Nematology 2, 17–30. doi: 10.1163/156854100508863

[B7] Forge T. A. MacGuidwi A. E. (1989). Nematode autofluorescence and its use as an indicator of viability. J. Nematol 21, 399–403. 19287626 PMC2618949

[B8] Holladay B. H. Willett D. S. Stelinski L. L. (2016). High throughput nematode counting with automated image processing. BioControl 61, 177–183. doi: 10.1007/s10526-015-9703-2

[B9] Huang J. Rathod V. Sun C. Zhu M. Korattikara A. Fathi A.. (2016). “Speed/accuracy trade-offs for modern convolutional object detectors,” in Proc. IEEE CVPR. (Honolulu, HI, USA: IEEE Conference on Computer Vision and Pattern REcognition (CVPR)). 3296–3297.

[B10] Hussey R. S. Barker K. R. (1973). A comparison of methods for collecting inocula for *Meloidogyne* spp. including a new technique. Plant Dis. Rep. 57, 1025–1028.

[B11] Jones J. T. Haegeman A. Danchin E. G. J. Gaur H. S. Helder J. Jones M. G. K.. (2013). Top 10 plant-parasitic nematodes in molecular plant pathology. Mol. Plant Pathol. 14, 946–961. doi: 10.1111/mpp.12057 23809086 PMC6638764

[B12] Kalwa U. Legner C. Wlezien E. Tylka G. Pandey S. (2019). New methods of removing debris and high-throughput counting of cyst nematode eggs extracted from field soil. PloS One 14, e0223386. doi: 10.1371/journal.pone.0223386 31613901 PMC6793949

[B13] Schomaker C. H. Been T. H. (1998). “Errors due to subsampling of soil samples with *Globodera rostochiensis* and *G. pallida*, and to other laboratory procedures,” in *Quantitative studies on the management of potato cyst nematode (Globodera* spp.*) in The Netherlands* . Eds. Been T. H. Schomaker C. H. (Wageningen University, Wageningen), 35–70.

[B14] Seinhorst J. W. (1988). The estimation of densities of nematode population in soil and plants. Vaxtskyddsrapporder 51, 107.

[B15] Shabrina N. H. Lika R. A. Indarti S. (2023). Deep learning models for automatic identification of plant-parasitic nematode. Artif. Intell. Agric. 7, 1–12. doi: 10.1016/j.aiia.2022.12.002

[B16] Uhlemann J. Cawley O. Kakouli-Duarte T. (2020). “Nematode identification using artificial neural networks,” in Proceedings of the 1st International Conference on Deep Learning Theory and Applications. 13–22 (Setubal, Portugal: SCITEPRESS-Science and Technology Publications, Lda). doi: 10.5220/0009776600130022

