# Supplementary figures and images for: Counting nematodes made easy: leveraging AI-powered automation for enhanced efficiency and precision
Source: Front Plant Sci. 2024 Jun 26;15:1349209. doi: 10.3389/fpls.2024.1349209 (PMC11238600; doi:10.3389/fpls.2024.1349209)

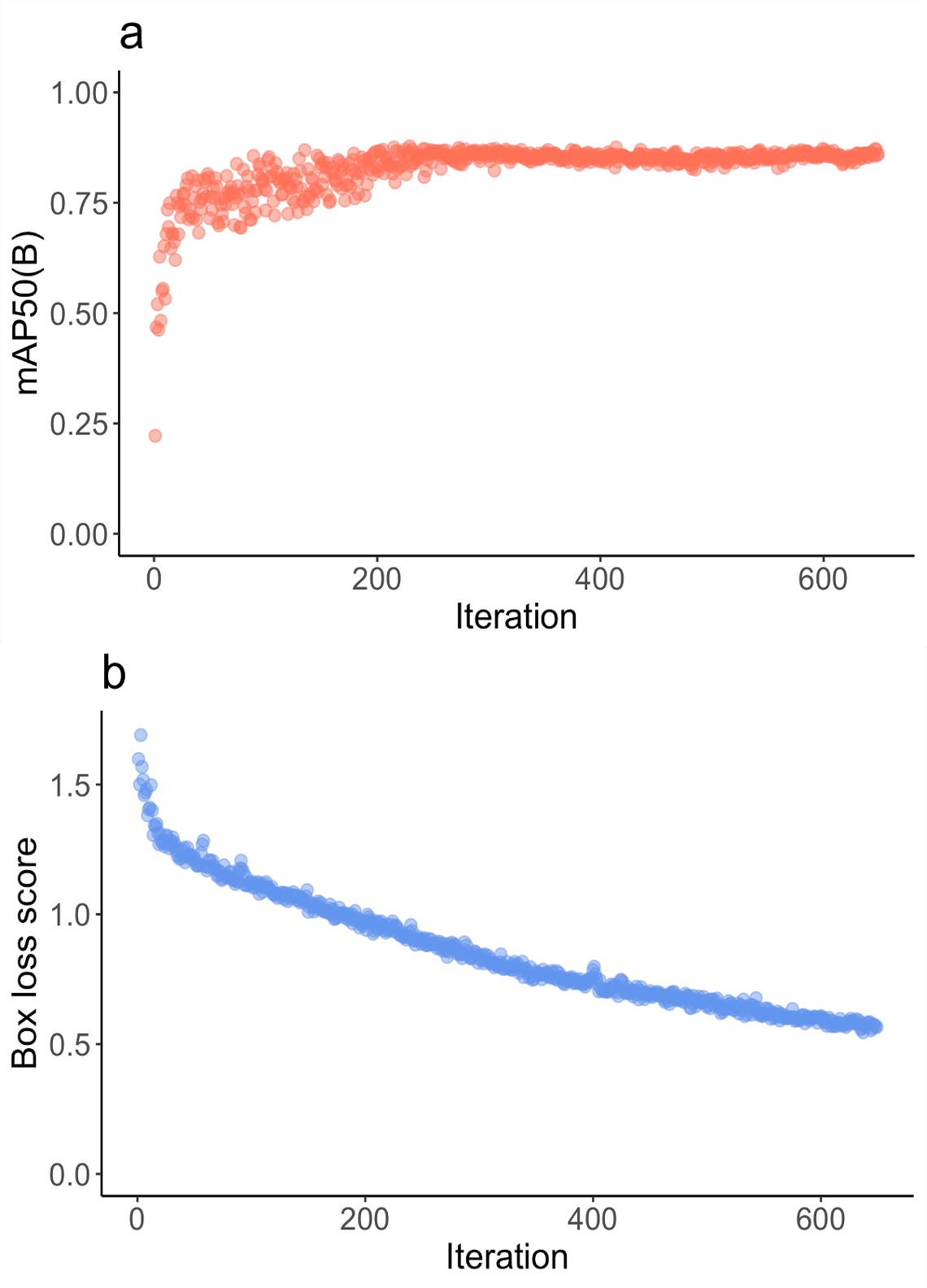

Supplement: Supplementary Figure 1 — Scores of mAP50(B) (A) and Box Loss (B) over iterations for the YOLOv8x deep-learning model for nematode egg detection. [file Image_1.tiff]

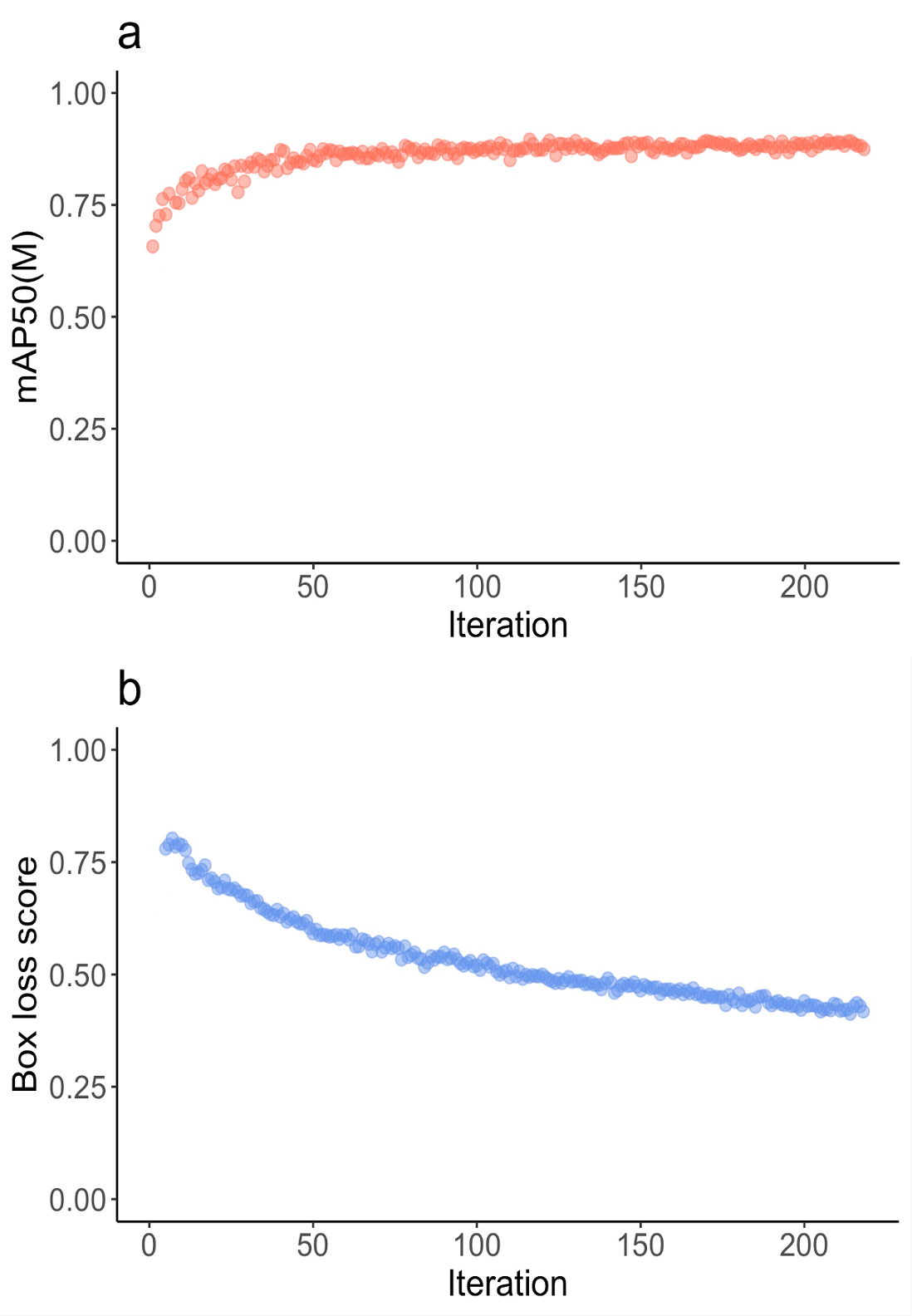

Supplement: Supplementary Figure 2 — Scores of mAP50(M) (A) and Box Loss (B) over iterations for the YOLOv8x deep-learning model for nematode juvenile detection. [file Image_2.tiff]
